# Noise-robust markerless video gait anomaly detection via two-stage acquisition and LSTM autoencoders

**DOI:** 10.1038/s41598-025-26169-9

**Published:** 2025-11-26

**Authors:** Hyerin Yoon, Eunah Jo, Seungjae Ryu, Jun-Il Yoo, Minkyeong Kim, Jin Hyun Kim

**Affiliations:** 1 Department of AI Convergence Engineering, Gyeongsang National University, 501, Jinju-daero, Jinju-si, Gyeongsangnam-do South Korea; 2 Department of Orthopaedic Surgery, Inha University Hospital, 27 Inhang-ro, Jung-gu, Incheon, South Korea; 3 Gyeongsang National University Hospital, 79, Gangnam-ro, Jinju-si, Gyeongsangnam-do South Korea

**Keywords:** Neurophysiology, Computational biology and bioinformatics, Engineering, Neurological disorders

## Abstract

We propose a markerless gait anomaly detection system for orthopedic screening. The framework combines MediaPipe-based joint tracking, unsupervised LSTM-autoencoder modeling, and targeted preprocessing to address clinical video noise. Trained only on normal gait, the model detects abnormal patterns in sarcopenia (SA) and Parkinson’s disease (PD) patients, achieving detection rates of 97% and 88%, respectively. Our method achieves state-of-the-art performance in sarcopenia detection, surpassing recent sensor-based approaches that require wearable devices or handcrafted features. Furthermore, to the best of our knowledge, this is the first unified markerless framework capable of identifying both sarcopenia and Parkinson’s disease using a single video-based system. To improve input quality, we address three common sources of error–frame imbalance, clothing interference, and background clutter—using YOLO-based frame filtering and semantic segmentation. This increased usable gait data by up to 38%. Joint-level analysis identified the knees as the most responsive to gait abnormalities, enabling interpretable and localized assessments. Our results highlight the potential of a scalable, non-invasive system for early detection and monitoring of musculoskeletal disorders.

## Introduction

Machine learning has become a vital tool in modern medicine, demonstrating strong performance in diagnosis, treatment planning, and drug development^1–3^. Decades of labeled medical data have enabled models to surpass traditional methods in disease detection, prognosis, and therapy design^4–6^. For instance, a machine learning system outperformed radiologists in mammogram analysis for breast cancer detection^7^, and deep learning has enhanced the accuracy of diabetic retinopathy diagnosis from retinal images^8^. These achievements highlight the potential of machine learning to advance personalized care and accelerate biomedical innovation.

In orthopedics, there is growing interest in using machine learning for gait analysis to evaluate musculoskeletal conditions, including osteoarthritis, spinal cord injury, and cerebral palsy^9–11^. Gait modeling methods fall into two main categories: marker-based and markerless. Marker-based systems are precise but invasive, time-consuming, and costly. In contrast, markerless systems—such as OpenPose and MediaPipe—estimate body poses directly from videos or images, offering a more scalable and non-intrusive solution^12–16^.

Recent studies have shown strong results in gait-based disease classification using supervised learning. For example, Kim et al. ^17^ reported 92–96% accuracy in sarcopenia detection by combining smart insoles with handcrafted pose features (e.g., *Hip_dif*, *Ankle_dif*) and classical classifiers like random forest and SVM. Likewise, He et al. ^18^ achieved 93% accuracy for Parkinson’s disease by reconstructing 3D poses from 2D joint trajectories and analyzing gait parameters. While effective, these methods depend heavily on labeled datasets, wearable sensors, and disease-specific annotations, which limit their scalability.

In this study, we propose a fully markerless and contact-free framework for detecting abnormal gait patterns associated with musculoskeletal disorders. Our goal is not to diagnose specific diseases, but to build a real-time system capable of identifying general gait anomalies. To ensure broader applicability, we use video data from patients with Parkinson’s disease and sarcopenia as representative cases. The proposed method combines MediaPipe-based joint tracking with an unsupervised LSTM-autoencoder trained only on normal gait data. This allows the model to detect deviations without the need for disease-specific labels or wearable devices. To enhance robustness, we introduce a noise-aware preprocessing pipeline targeting three common environmental issues: frame imbalance (addressed using YOLO-based selection), clothing artifacts (standardized attire), and background interference (semantic segmentation). These steps increased usable gait data by 38% for normal subjects, 33% for sarcopenia, and 2% for Parkinson’s disease, improving model reliability.

The model achieved detection accuracies of 97% for sarcopenia and 88% for Parkinson’s disease, meeting or exceeding current supervised methods. Joint-level analysis also identified the knees as the most sensitive indicators of gait abnormalities, providing clinical insight and supporting use in real-world settings.

The main contributions of this work are as follows:

- We identify three major sources of environmental noise in markerless gait tracking—frame imbalance, clothing-induced artifacts, and background interference—and propose practical strategies to mitigate their impact.
- We develop a two-stage data acquisition and preprocessing strategy that incorporates YOLO-based subject-capturing optimization and semantic segmentation to reduce environmental noise.
- We implement and validate an LSTM-autoencoder model that achieves high detection rates of 97% for SA and 88% for PD, surpassing existing markerless methods in sarcopenia detection.

Our findings advance the field of gait-based orthopedic diagnosis by offering a practical, non-invasive framework that integrates robust preprocessing with deep learning-based anomaly detection. By conducting both quantitative performance evaluation and clinical interpretation of joint-level anomalies, our work contributes to the development of more robust, generalizable, and deployable gait analysis tools in orthopedic and neurological care.

## Results

This section evaluates the performance of the proposed anomaly detection framework in identifying abnormal gait patterns in patients with sarcopenia (SA) and Parkinson’s disease (PD). We assess detection accuracy across five joint regions: right hip, left hip, right knee, left knee, and nose–shoulder. Joint-level sensitivity is analyzed to identify the most critical regions for detecting pathological gait. In SA, gait abnormalities are primarily caused by muscle loss^19^, while in PD, they result from stiffness and reduced mobility^20^. In this work, a subject’s gait is classified as abnormal if any of the five joints exceed the anomaly threshold.

### Anomaly detection results

**Fig. 1:**
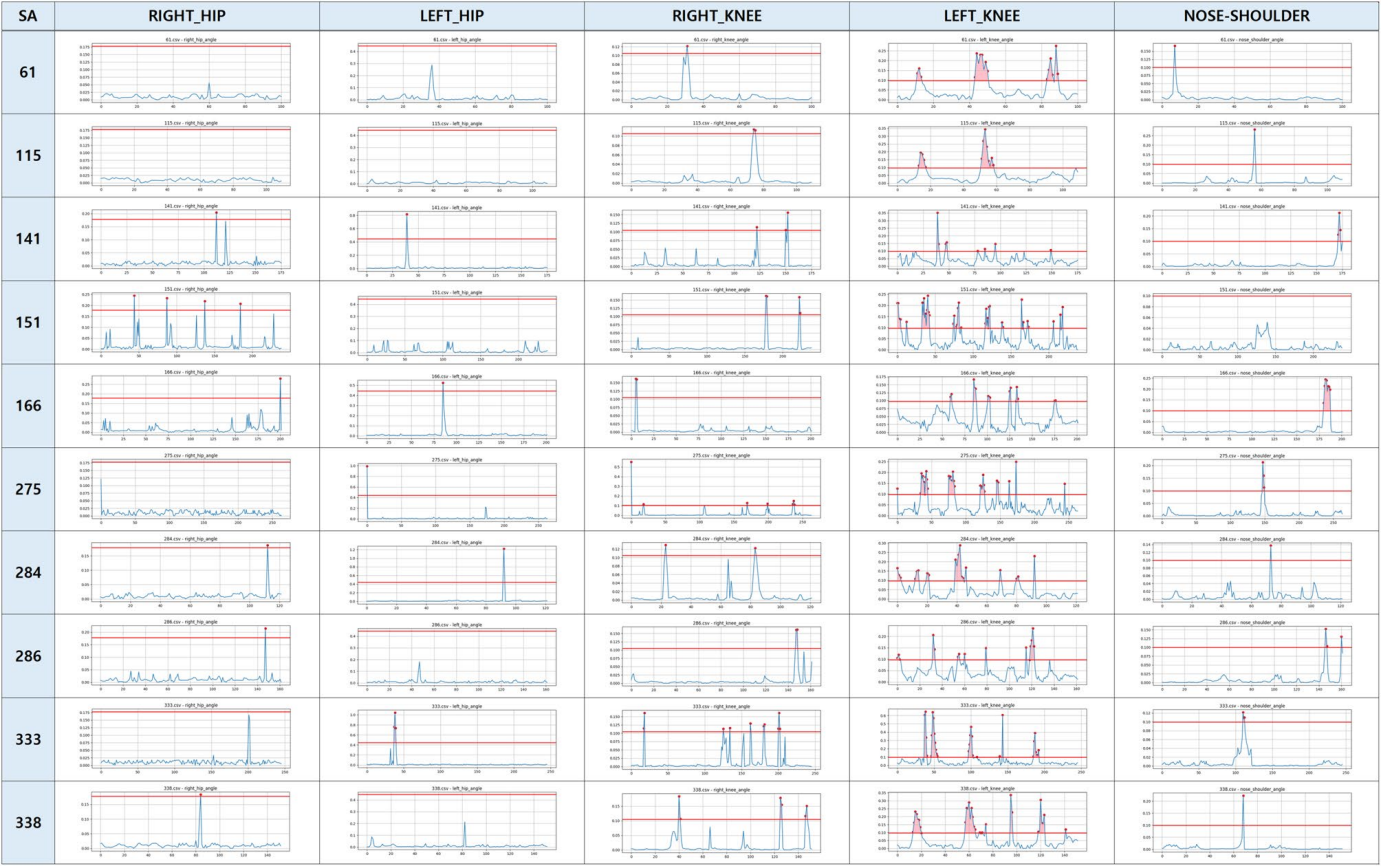
The results of anomaly gait detection for five joint parts of SA.

**Fig. 2:**
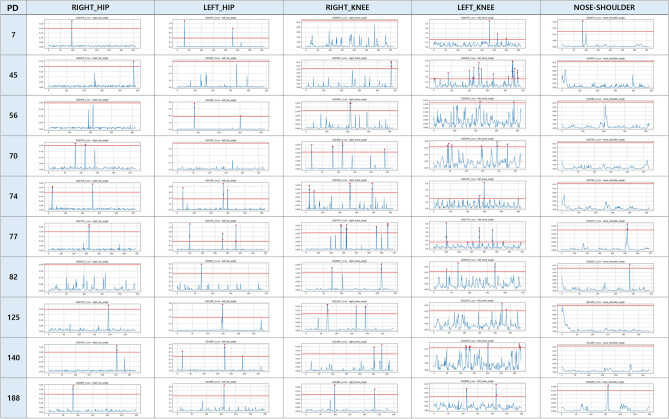
The results of anomaly gait detection for five joint parts of PD.

Figures 1 and 2 present anomaly detection results for sarcopenia (SA) and Parkinson’s disease (PD) patients. Each graph lists patient IDs vertically and five joint regions horizontally: right hip, left hip, right knee, left knee, and nose–shoulder. For each joint, the reconstruction error is plotted over video frames. The red line indicates the anomaly threshold, and red dots denote detected outliers. Thresholds were defined individually for each joint model, based on average reconstruction error from the normal gait data. For example, patient ID 61 (SA) showed anomalies in the right knee, left knee, and nose–shoulder. In contrast, patient ID 77 (PD) exhibited abnormalities across all five joints, suggesting more widespread motor impairment.

These results show that knee anomalies are common in both SA and PD, while PD patients display broader joint involvement. This highlights the diagnostic importance of lower-limb joints and supports the use of joint-level patterns for disease-specific gait analysis.

**Fig. 3:**
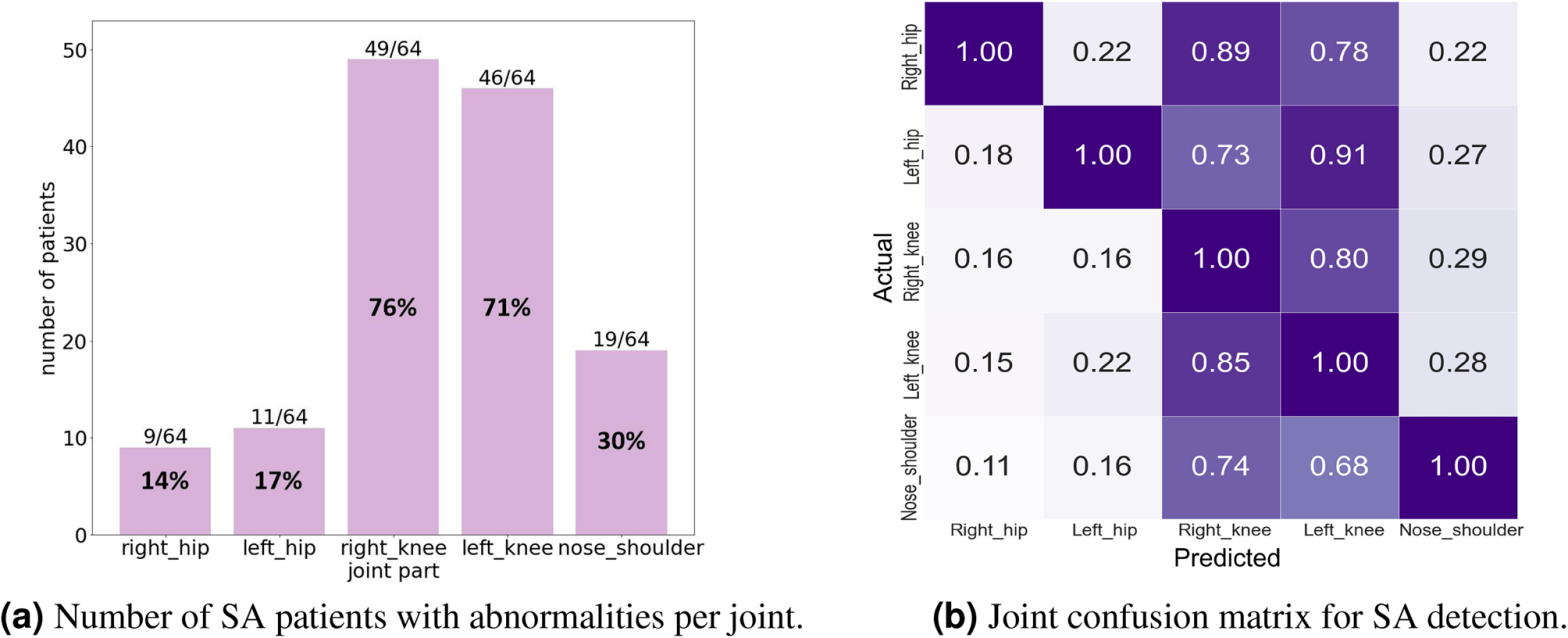
Summary of abnormal gait detection in SA patients: (**a**) number of patients per joint, (**b**) joint confusion matrix.

### Detection performance summary

Using the proposed anomaly detection classifier, 62 out of 64 sarcopenia (SA) patients (approximately 97%) and 43 out of 49 Parkinson’s disease (PD) patients (approximately 88%) were correctly identified as exhibiting abnormal gait. Based on these results, Table 1 summarizes the detection performance metrics, including accuracy, recall, precision, and F1 score for each disease group. Importantly, a subject is classified as having abnormal gait if *at least one of the five joints*—right hip, left hip, right knee, left knee, or nose–shoulder—*exceeds the predefined anomaly threshold*. This joint-wise thresholding strategy ensures high sensitivity by flagging localized deviations, even when other joints appear normal. As a result, the framework supports early and robust screening for gait disorders with diverse anatomical manifestations.

**Table 1:** Detection performance of the proposed gait anomaly detection model (recall and accuracy only).

Disease	Accuracy	Recall
Sarcopenia (SA)	97%	96.9%
Parkinson’s disease (PD)	88%	87.8%

We evaluated the performance of the proposed gait anomaly detection model on sarcopenia (SA) and Parkinson’s disease (PD) patients. As shown in Table 1, the model achieved an accuracy of 97% and recall of 96.9% for SA patients, and 88% accuracy with 87.8% recall for PD patients. These results confirm that the model successfully detects abnormal gait patterns in both disease groups. Since our evaluation primarily targeted pathological gait, the dataset included a limited number of normal cases. Consequently, metrics that depend on the number of false positives or true negatives, such as precision and F1 score, are excluded to avoid potential bias or instability in performance interpretation.

### Joint-wise anomaly detection analysis

Figure 3ashows the number of sarcopenia (SA) patients with detected anomalies at each joint, based on a uniform dataset of 64 patients. The right and left knees were the most frequently affected, with 76% (49/64) and 71% (46/64) of patients showing anomalies, respectively. These results underscore the diagnostic value of knee joints as primary indicators of abnormal gait in SA. In contrast, the hip joints exhibited lower sensitivity (14% for right hip and 17% for left hip), while the nose–shoulder region showed a moderate detection rate of 30%, suggesting a secondary role in gait characterization. These findings support our joint-wise evaluation strategy and validate the emphasis on lower-body joints in clinical assessment.

Figure 3bpresents the joint confusion matrix, indicating the co-occurrence of anomalies across joints. When the right knee showed an anomaly, the right hip was also detected as abnormal in 89% of such cases. The right and left knees had the highest average co-detection rates (80.25% and 79.25%), whereas the hips and nose–shoulder showed relatively low co-detection (15–26.5%). These patterns further confirm the central role of the knees in SA-related gait impairment and suggest potential biomechanical correlations or pose estimation limitations in detecting anomalies at the hip and upper-body regions.

Clinically, these results highlight the need to prioritize knee joint analysis in SA diagnosis and monitoring. Improving upper-body joint tracking—particularly in the nose–shoulder region—may offer additional value in future gait anomaly studies.

**Fig. 4:**
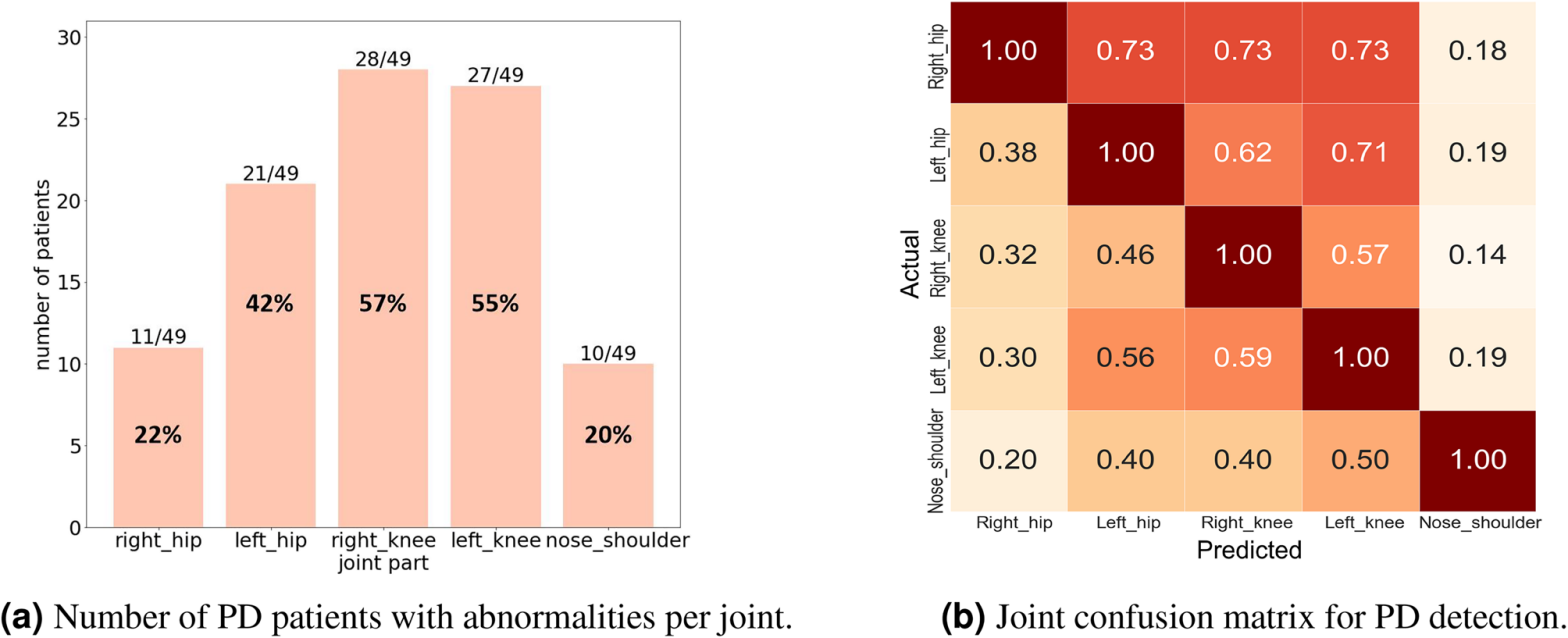
Summary of abnormal gait detection in PD patients: (**a**) number of patients per joint, (**b**) joint confusion matrix.

Figure 4apresents the joint-wise anomaly detection results for 49 Parkinson’s disease (PD) patients. Among the 43 patients flagged as abnormal (88%), the right and left knees were the most frequently contributing joints, with detection rates of 57% (28/49) and 55% (27/49), respectively. This underscores the knees as key indicators in PD gait analysis. Hip joints followed, while the nose–shoulder region showed the lowest detection contribution, further supporting the focus on lower-body joints in our framework. Figure 4bpresents the joint confusion matrix, illustrating co-detection rates across joints. The right and left knees demonstrated the highest average co-detection rates (58.5% and 62.75%, respectively), followed by the hips (30% right, 53.75% left), and the nose-shoulder region (17.5%). These patterns suggest that knee abnormalities are not only common but also strongly interconnected with other joint anomalies, reinforcing their diagnostic importance in PD gait analysis. The relatively lower detection rates in the hips and nose-shoulder may reflect biomechanical differences in PD gait or limitations in the pose estimation algorithm. We evaluated the anomaly detection performance for each of the five joints—right hip, left hip, right knee, left knee, and nose–shoulder—across both sarcopenia (SA) and Parkinson’s disease (PD) groups.

**Table 2:** Joint-wise performance metrics: true positive (TP), false negative (FN), false positive (FP), true negative (TN), accuracy, precision, recall, and F1 score.

Joint	TP	FN	FP	TN	Accuracy (%)	Precision (%)	Recall (%)	F1 score (%)
Right hip	3	0	93	20	19.83	3.13	100.00	6.06
Left hip	3	0	81	32	30.17	3.57	100.00	6.90
Right knee	2	1	36	77	68.10	5.26	66.67	9.76
Left knee	3	0	40	73	65.52	6.98	100.00	13.04
Nose–shoulder	1	2	84	29	25.86	1.18	33.33	2.27

TP and FN refer to cases where the subject has sarcopenia (SA) or Parkinson’s disease (PD), i.e., positive samples. FP and TN refer to normal (negative) cases

Table 2 reports joint-wise evaluation metrics, including accuracy, precision, recall, and F1 score. The results show that the knee joints—particularly the left knee—yield relatively higher values across all performance indicators, with a recall of 100% and F1 score of 13.04%. The right knee also demonstrates strong performance, with the highest accuracy (68.10%) among all joints. In contrast, the hip and nose–shoulder joints exhibit notably lower precision and F1 scores due to a high number of false positives.

These observations reflect the challenge of joint-wise anomaly detection, particularly when normal samples are limited. In this study, any anomaly detected in one or more joints results in the subject being classified as abnormal. As a result, even joints with high false positive rates (e.g., right hip: FP = 93) do not necessarily reduce subject-level detection performance. This explains why the overall accuracy remains high—97% for sarcopenia and 88% for Parkinson’s disease—despite the low precision scores at the joint level.

These findings suggest that while individual joint detectors may show varying levels of precision, their collective use supports sensitive detection of pathological gait. The framework is particularly effective in detecting subtle or localized deviations, and future work will focus on reducing false positives in low-discriminative joints to further enhance reliability.

Traditional ROC curve analysis was not used in this study due to significant class imbalance in our dataset—29 normal subjects versus 113 patients (64 SA and 49 PD). Since ROC metrics can overestimate performance in such imbalanced settings, we instead used precision, recall, and F1-score, which provide more reliable evaluation for our anomaly detection models.

Clinically, these findings underscore the importance of prioritizing knee monitoring in PD diagnosis and highlight the potential value of enhancing upper-body detection methods to improve comprehensive gait assessment.

Analysis of the anomaly detection results for both SA and PD across the five joint parts reveals that anomalies in the knees show a particularly high detection rate. This is due to the more pronounced joint angle changes in the knees compared to other joint parts, making them critical for detecting gait abnormalities. Additionally, when comparing hip joint anomaly detection results, PD patients show a higher anomaly detection rate than SA patients.

This discrepancy indicates that hip joint movement variations are more pronounced in PD patients, leading to a higher anomaly detection rate in this area. Regarding anomaly detection results for the nose-shoulder region, both SA and PD patients exhibit lower detection rates for gait abnormalities.

The reduced detection rate in this region might be attributed to the training data including normal pedestrians walking with their heads down. This limitation in the dataset likely impaired the model’s capability to effectively recognize abnormal nose-shoulder movements indicative of atypical gait. These findings highlight the significance of accounting for specific joint characteristics and potential limitations in training data when interpreting anomaly detection outcomes, stressing the necessity for further validation across varied scenarios.

Certain joints, such as the nose–shoulder region, showed relatively high false alarm rates and lower anomaly detection sensitivity. These joints are less biomechanically involved in gait propulsion and support compared to lower limb joints like the knees and hips^21^. In particular, the nose–shoulder joint is susceptible to variability due to head posture. For example, in our training data, some normal subjects walked while looking downward, leading the model to learn forward neck flexion as a normal pattern. Consequently, neck flexion in test subjects—common in PD patients—was less likely to be flagged as abnormal. This suggests that improvements in training data collection (e.g., enforcing consistent forward gaze) could enhance model sensitivity for upper body joints.

### Statistical comparison of joint angles using paired *t*-test and Cohen’s *d*

To statistically validate the differences in joint angle trajectories between normal and patient groups, we conducted paired *t*-tests for each joint. The null hypothesis ($H_0$) assumes no difference between the groups, and a *p*-value less than 0.05 indicates a statistically significant difference.

Table 3 summarizes the *p*-values for comparisons between control subjects and the sarcopenia (SA) and Parkinson’s disease (PD) groups.

**Table 3:** Paired t-test *p*-values for joint angles between control and patient groups. Statistically significant results ($p < 0.05$) are highlighted in bold.

Comparison	Right hip	Left hip	Right knee	Left knee	Nose–shoulder
Normal vs. sarcopenia	0.93061	0.28282	**0**	**0**	0.34655
Normal vs. Parkinson’s	0.74076	0.44422	**0.00046**	**0**	**0.00697**

A *p*-value less than 0.05 indicates a statistically significant difference.

Knee joints showed statistically significant differences in both disease groups, aligning with their known clinical relevance and high anomaly detection rates in our model. For PD patients, significant differences were also found in the nose–shoulder joint, possibly reflecting upper-body posture changes such as trunk rigidity and reduced arm swing.

To quantify the effect size of each joint, we calculated Cohen’s *d*, which measures standardized group differences. Effect sizes greater than 0.8 are considered large.

Knees consistently showed the largest effect sizes (*d*$> 0.9$) and the most significant *p*-values, as shown in Table 4, reinforcing their role as the most discriminative joints for gait anomaly detection. The nose–shoulder region showed moderate effect size in PD (*d* = 0.566), suggesting auxiliary diagnostic potential. In contrast, the hips yielded small effect sizes (*d*$< 0.2$), indicating limited contribution.

**Table 4:** Cohen’s *d* effect size comparison across joint parts.

Group	Right hip	Left hip	Right knee	Left knee	Nose–shoulder
Normal / SA	0.103	0.144	**1.248**	**0.947**	0.123
Normal / PD	0.159	0.108	**0.931**	**1.353**	**0.566**

Cohen’s *d* values: 0.2 = small, 0.5 = medium, 0.8 = large effect size.

Overall, these statistical findings are consistent with our model’s joint-wise detection results and further emphasize the diagnostic value of knee joint dynamics in both sarcopenia and Parkinson’s disease.

## Data and methods

### Study design

The protocol for this retrospective study was approved by the Institutional Review Board of Gyeongsang National University Hospital in accordance with the Declaration of Helsinki. Due to the study’s retrospective design, the Institutional Review Board (GNUH 2022-01-032) waived the requirement for informed patient consent.

### Detection methods

This section provides an overview of MediaPipe and describes the data used to analyze orthopedic conditions. It also summarizes the preprocessing steps and the deep-learning model employed for anomaly detection.

MediaPipe^22^ is an open-source framework for human pose detection and tracking, comparable to Google’s OpenPose^23^. It enables real-time detection of facial, hand, and body joints in images or videos. MediaPipe is selected based on practical requirements for clinical deployment, including real-time processing capability (over 30 FPS on CPU), reliable estimation of key joints such as the hip and knee, and lightweight model size suitable for embedded or hospital systems. In our experiments, MediaPipe maintained balanced performance across static and dynamic gait conditions, including sarcopenia and Parkinson’s disease. Furthermore, its seamless integration with Python enables efficient coupling with time-series anomaly detection modules, facilitating clinical translation.

Table 5 compares MediaPipe and OpenPose in terms of the number of detected joint parts. Notably, MediaPipe detects a greater number of facial and body landmarks, providing finer granularity for movement analysis.

**Table 5:** Comparison of joint parts detected by MediaPipe and OpenPose.

	OpenPose	MediaPipe
Hand	21	21
Face	70	468
Body	25	33

**Fig. 5:**
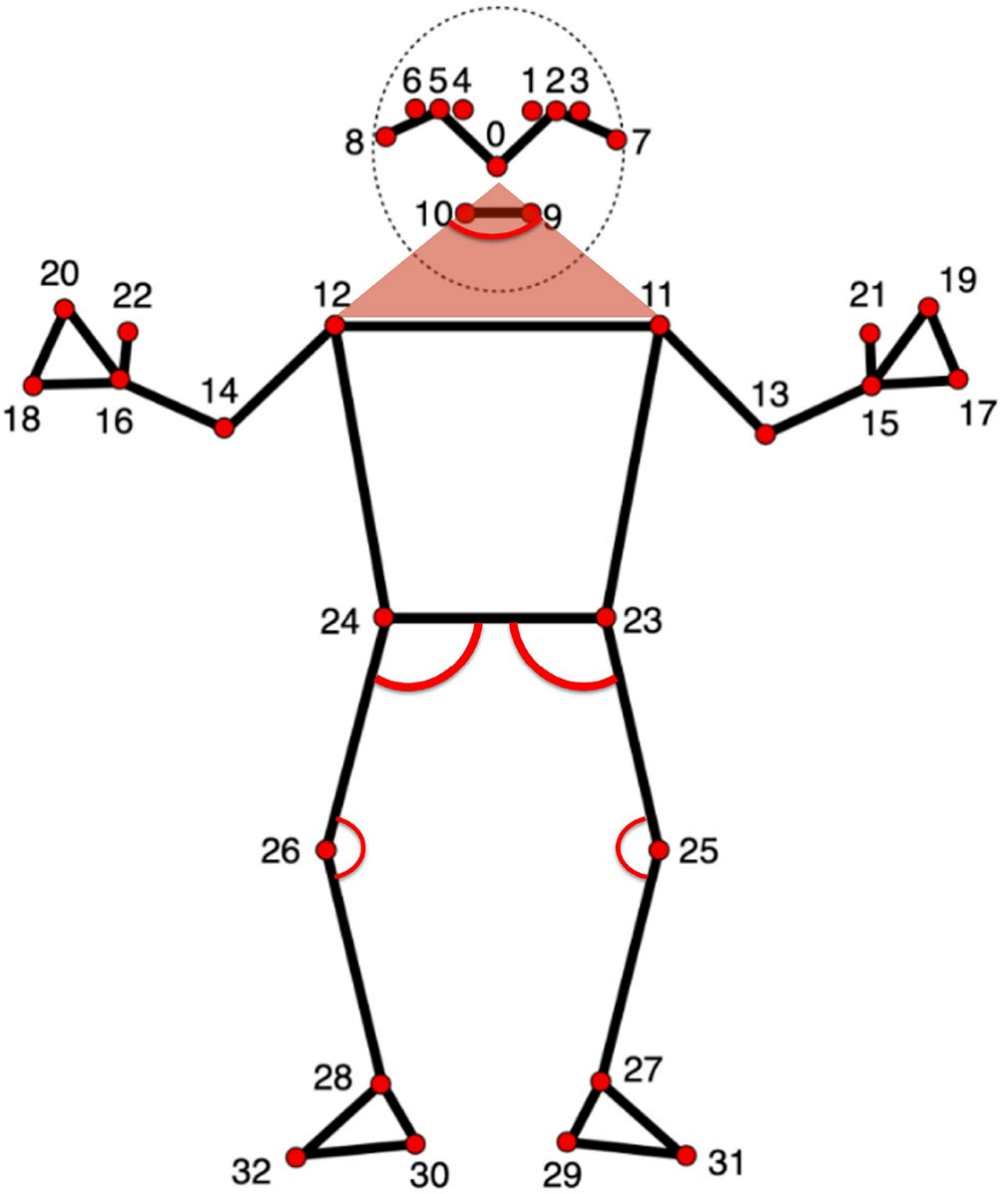
Extraction of five joint angles from MediaPipe output.

While MediaPipe offers a detailed representation of body movements, this study focused specifically on five joint angles—right and left knees, right and left hips, and the nose-shoulder angle—considered most relevant for gait anomaly detection in sarcopenia (SA) and Parkinson’s disease (PD). Figure 5 illustrates the extraction of these angles from MediaPipe outputs.

The decision to focus on these specific joints was guided by prior evidence indicating their critical role in distinguishing normal from pathological gait patterns. Although MediaPipe enables comprehensive full-body tracking, narrowing the analysis to key joints allowed the development of a more targeted and interpretable anomaly detection model.

### Datasets and preprocessing

This study focused on identifying abnormal gait patterns in patients with sarcopenia (SA) and Parkinson’s disease (PD). Abnormal gait in SA typically results from muscle loss^19^, whereas in PD it is characterized by muscle stiffness and slowed movement^20^. Abnormal gait was defined as the presence of anomalies in any of five body regions, acknowledging that both conditions can affect multiple joints.

Lateral gait videos were collected from the Orthopedic Department of Gyeongsang National University Hospital. Videos averaged 20 seconds in length at 30 frames per second (fps). The dataset included patients across a wide severity range, including those unable to walk properly. For example, patient A completed 10 steps in 20 s, whereas a more severe patient B managed only 3 steps in the same duration. This variability was intentionally preserved, as uniformly adjusting video length would risk excluding severe cases and biasing the analysis.

After quality checks, 29 out of 30 normal gait videos were retained, with one excluded due to MediaPipe recognition errors. Among SA patients, three redundant videos were removed from an initial 120, resulting in 117 for analysis. For PD patients, 11 out of 60 videos were excluded due to recognition errors, yielding 49 usable videos. Notably, the exclusion rate was higher in the PD group, likely reflecting greater movement irregularity and technical detection challenges. Normal gait data were defined based on visual inspection and confirmation by orthopedic specialists, ensuring the absence of abnormal movement patterns.

**Fig. 6:**
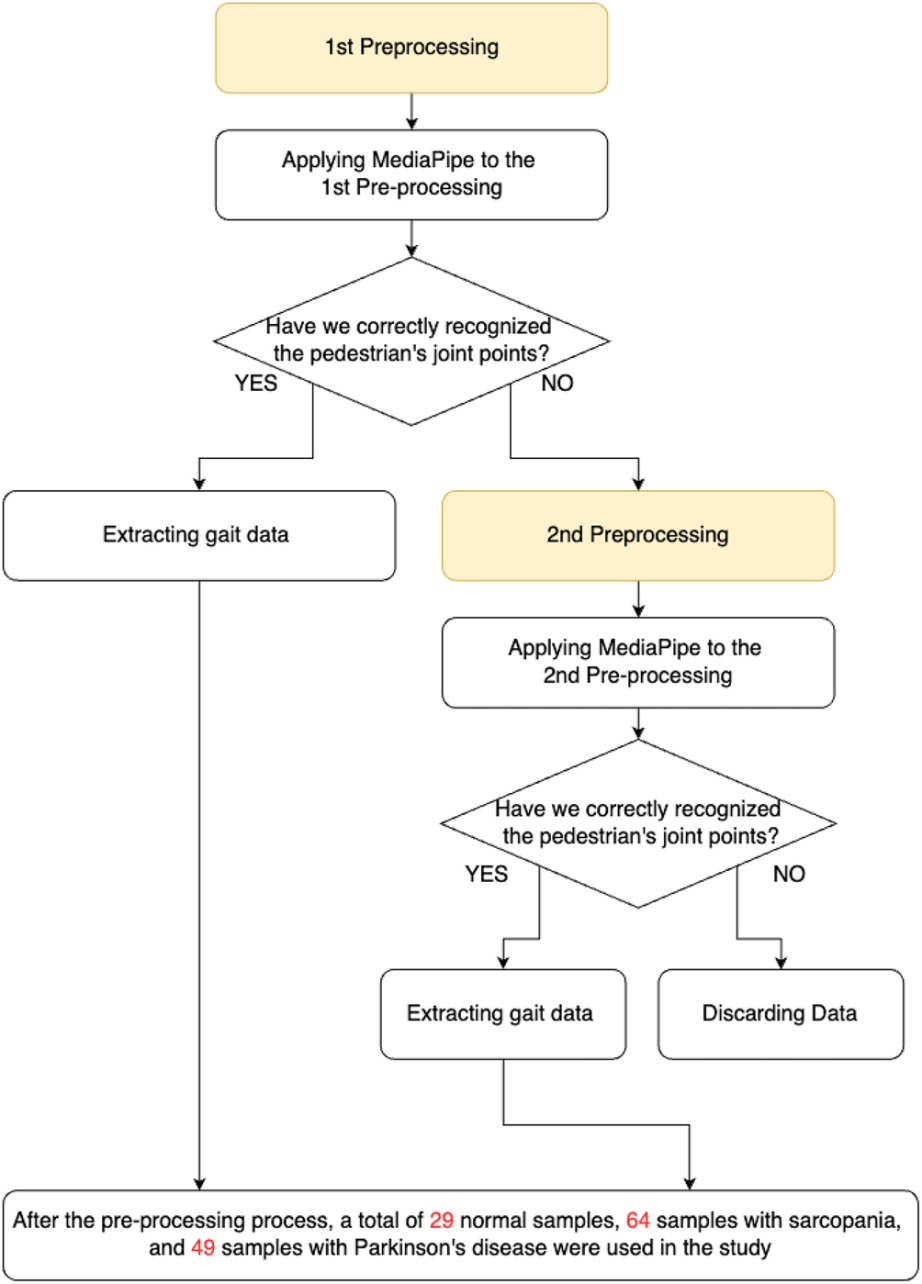
Preprocessing for gait data extraction.

Figure 6 illustrates the preprocessing workflow for gait data extraction. Two main preprocessing stages were employed to ensure accurate joint detection using MediaPipe.

The first preprocessing stage involved removing video frames that caused recognition errors, focusing specifically on lateral body views. Frames where the participant turned or where joints were occluded by the environment were excluded to ensure clear visibility of the body outline.

In the second preprocessing stage, for videos that initially failed joint recognition, background segmentation was applied to isolate the pedestrian. MediaPipe was then reapplied to these segmented frames. If joint detection succeeded after segmentation, the gait data were extracted; otherwise, the video was discarded.

Importantly, this manual preprocessing was necessary due to variations in recording conditions, including location, camera angle, and subject-to-camera distance, which complicated automation. After completing the preprocessing pipeline, a total of 29 normal, 64 sarcopenia, and 49 Parkinson’s disease samples were retained for analysis, as shown in the figure.

**Fig. 7:**
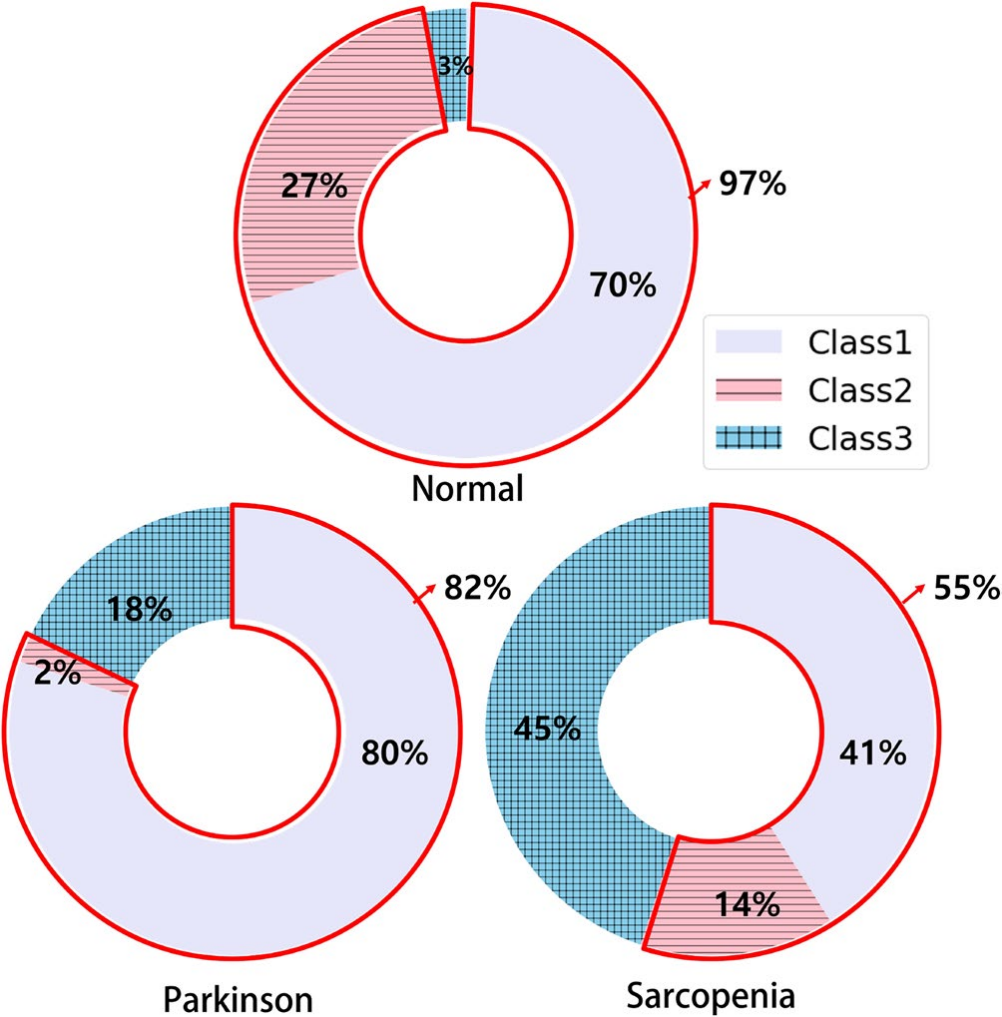
Video data classified into three categories.

The gait videos used in MediaPipe were categorized into three classes, as illustrated in Fig. 7. This classification was implemented to manage data quality, optimize preprocessing workflows, and ensure consistency in subsequent analyses.

- Class 1 includes videos successfully processed after the first stage of preprocessing, as shown in Fig. 6.
- Class 2 includes videos that required additional background masking during the second preprocessing stage before successful processing by MediaPipe.
- Class 3 consists of videos excluded from analysis due to persistent recognition failures caused by a combination of factors, including frame imbalance, clothing-induced blurring, body skeleton misidentification, the use of walking aids (e.g., canes), and the presence of assistant caretakers.

Notably, Class 3 highlights practical challenges encountered when applying automated pose estimation tools in clinical populations, pointing to the need for improved algorithms or controlled recording environments. A summary of the sample distribution across these classes is provided in the results section.

### Abnormal gait detection for orthopedic conditions using LSTM-autoencoder

This section describes the use of an LSTM-autoencoder, a deep learning-based anomaly detection method, for identifying abnormal gait patterns associated with orthopedic conditions. Long Short-Term Memory (LSTM) networks^24^ are designed to overcome the vanishing gradient problem in recurrent neural networks (RNNs), enabling the retention of long-term dependencies in time series data. Autoencoders, as unsupervised learning models, compress input data into a latent representation and reconstruct it back to its original form. An LSTM-autoencoder^25^ integrates LSTM layers in both the encoder and decoder to model sequential patterns and detect deviations.

In this study, an LSTM-autoencoder was employed to model the temporal dynamics of joint angle sequences derived from gait data. The model was trained exclusively on normal gait samples, with the assumption that it would reconstruct these sequences with low error. Conversely, abnormal gait patterns—such as those associated with sarcopenia (SA) or Parkinson’s disease (PD)—were expected to yield significantly higher reconstruction errors due to their deviation from the learned normal patterns.

To determine whether a given gait sequence is anomalous, we calculated the mean squared error (MSE) between the input and the reconstructed sequence. A sample was flagged as anomalous if its MSE exceeded a threshold defined as the mean plus two standard deviations ($\mu + 2\sigma$) of the reconstruction errors observed in the training (normal) set. This thresholding strategy is grounded in classical statistical theory, where values beyond $\mu + 2\sigma$ are typically considered outliers under the assumption of a normal distribution.

This approach offers a simple yet effective way to detect deviations without requiring disease-specific labels. Similar thresholding strategies have been widely adopted in prior anomaly detection research, including autoencoder-based models^26^ and LSTM-based sequence anomaly detection^27^. We chose this method due to its reproducibility, low computational cost, and ease of implementation in clinical scenarios. Future work may explore adaptive or distribution-free thresholding techniques to further improve sensitivity and specificity.

The choice of LSTM-autoencoder over classical methods, such as Mahalanobis distance-based approaches, was motivated by its superior ability to capture nonlinear temporal relationships, which are critical in modeling complex gait patterns. Previous applications have demonstrated its high performance in various domains, including air quality monitoring^28^, weather anomaly detection^29^, arrhythmia analysis^30^, and ECG abnormality detection^31^.

Separate LSTM-autoencoder models were trained for five joint angles (right and left knees, right and left hips, and nose-shoulder) extracted from normal subjects. The established thresholds were then used to evaluate SA and PD patients. This approach allowed the identification of joint-specific gait anomalies, providing interpretable insights into the biomechanical patterns associated with these conditions.

Importantly, this framework highlights the clinical relevance of automated gait anomaly detection and emphasizes the need for reproducibility. Hyperparameters and threshold selection procedures were standardized across experiments, ensuring consistency. While the LSTM-autoencoder is inherently a black-box model, the use of joint-specific training and thresholding provides an interpretable layer, enhancing its applicability in clinical decision support.

### Model architecture and training

**Fig. 8:**
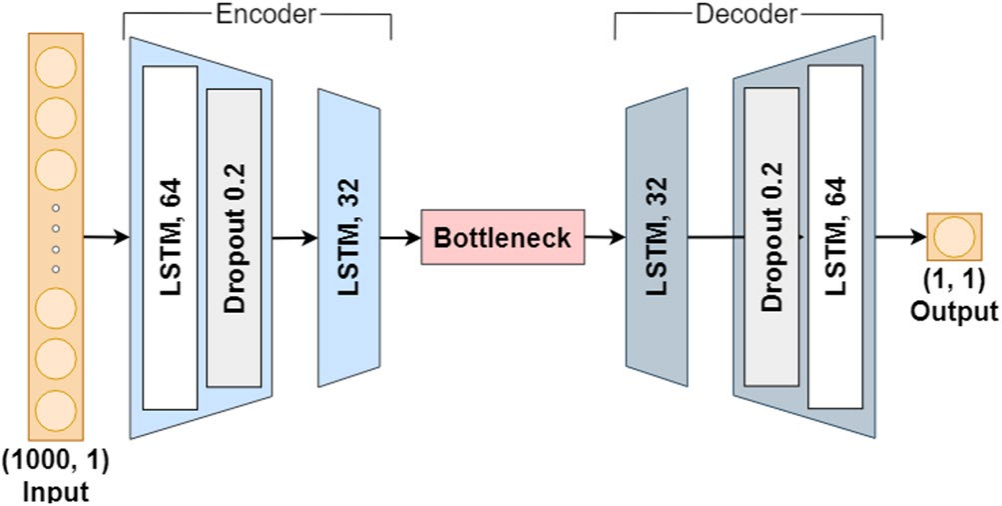
LSTM-autoencoder architecture used for all joints. For the right knee, the encoder-decoder configuration uses LSTM layers with 64–32 and 32–64 units, respectively; for other joints, the configuration uses 32–16 and 16–32 units.

**Table 6:** Hyper-parameters: RH(right hip), LH(left hip), RK(right knee), LK(left knee), NS(nose shoulder).

	RH	LH	RK	LK	NS
Optimizer	Adam	Adam	Adam	Adam	Adam
Epoch	200	150	150	200	150
Batch size	16	32	32	64	64
Learning rate	0.0001	0.0001	0.0001	0.0001	0.0001

Figure 8 illustrates the unified LSTM-autoencoder architecture used for all joints in the study. The architecture consists of an encoder with two LSTM layers and a decoder with two LSTM layers, connected by a bottleneck layer that compresses the input sequence into a 16-dimensional latent space. This bottleneck enables the model to capture essential temporal patterns while minimizing redundant information. Notably, the encoder-decoder configuration uses LSTM layers with 64 and 32 units, and 32 and 64 units, respectively, for the right knee; for the other joints (right and left hips, left knee, and nose-shoulder), the configuration uses 32-16 units in the encoder and 16-32 units in the decoder.

The LSTM-autoencoder models were trained using 26 normal gait samples per joint, with hyperparameters summarized in Table 6. These hyperparameters, including optimizer, epoch count, batch size, and learning rate, were determined through grid search optimization to balance model capacity and generalization performance. Dropout layers with a rate of 0.2 were applied in both the encoder and decoder to mitigate overfitting.

Performance evaluation was conducted on 64 lateral gait videos from sarcopenia (SA) patients and 49 videos from Parkinson’s disease (PD) patients. Reconstruction error, measured as mean squared error (MSE) between input and output sequences, served as the primary anomaly detection metric. Detection thresholds were defined per joint as the mean plus two standard deviations of reconstruction errors in the normal gait data. Table 7 presents the experimental results, including the false alarm rates observed in normal gait, providing a baseline for evaluating the detection models.

From a clinical perspective, this architecture enables joint-specific anomaly detection while maintaining interpretability, particularly in the knee joints, which play a pivotal role in distinguishing pathological from normal gait. The explicit reporting of architectural details and hyperparameter settings ensures reproducibility and facilitates the adoption of this approach in future clinical gait analysis applications.

**Table 7:** False alarm rate using 5-folds for normal data.

	1	2	3	4	5	SUM	AVG
RH	0	1	1	1	0	3	10%
RK	0	0	0	1	1	2	7%
LH	0	3	0	0	1	4	13%
LK	1	0	2	3	2	8	27%
NS	2	2	5	3	1	13	44%

The model was validated using five-fold cross-validation on normal gait data, as summarized in Table 7. The right hip and right knee exhibited relatively low false alarm rates, under 10%, indicating stable model performance in these joints. In contrast, the left knee and nose-shoulder regions showed higher false alarm rates, at 27% and 44%, respectively. These elevated rates may reflect greater inter-subject variability in gait asymmetry and upper-body movements, which pose challenges for consistent joint tracking and anomaly detection.

From a clinical perspective, reducing false alarms in these regions is critical to avoid unnecessary clinical follow-up and ensure the reliability of automated gait assessment tools. To address this, future work will explore strategies such as joint-level threshold optimization, data augmentation, and the integration of multi-joint prediction models to improve robustness in these challenging anatomical regions. Importantly, the five-fold design ensured balanced evaluation across the dataset, enhancing the generalizability of the findings.

### Ablation study: comparison of joint estimation models

To evaluate the impact of joint estimation quality on downstream anomaly detection, we compared multiple pose estimation models—including YOLO-Pose, MoveNet, OpenPose, and MediaPipe—using the same sarcopenia (SA) and Parkinson’s disease (PD) patient video dataset. The goal of this comparison is to assess which model offers the most reliable joint tracking under diverse gait patterns and recording conditions. We focused on the average joint estimation accuracy across two disease groups to ensure consistency and robustness in temporal anomaly detection.

**Table 8:** Joint-wise accuracy comparison across different pose estimation models.

Model	Points	Disease	Right hip	Left hip	Right knee	Left knee	Nose–shoulder	Total	Mean
YOLO	17	SA	20%	16%	55%	33%	8%	77%	81%
		PD	41%	27%	76%	16%	0%	86%	
MoveNet	17	SA	58%	47%	44%	47%	25%	83%	85%
		PD	16%	43%	31%	59%	51%	88%	
MediaPipe	33	SA	14%	17%	77%	72%	30%	97%	**92%**
		PD	22%	43%	57%	55%	20%	88%	
OpenPose	25	SA	67%	69%	69%	80%	13%	98%	90%
		PD	39%	24%	59%	31%	29%	82%	

As shown in Table 8, *MediaPipe* achieved the highest overall mean accuracy (92%) across both sarcopenia and Parkinson’s disease cases. While *OpenPose* recorded the highest accuracy for sarcopenia (98%), its performance for Parkinson’s disease dropped to 82%, indicating a larger inter-disease variance. In contrast, MediaPipe maintained stable accuracy for both sarcopenia (97%) and Parkinson’s disease (88%), demonstrating strong generalizability to different musculoskeletal conditions. Furthermore, MediaPipe showed particularly high accuracy for lower-body joints—especially the knees—whose precise estimation is critical for downstream time-series anomaly detection. These findings support the selection of MediaPipe as the backbone for joint estimation in our framework.

## Environmental challenges and optimization strategies for using MediaPipe

Markerless gait tracking systems, like MediaPipe, are more susceptible to noise compared to marker-based systems as they do not require markers to be attached directly to the body for data extraction. Such noise can impact the detection of abnormal gait using MediaPipe, with sources including frame imbalance, clothing, and backgrounds. Section outlined certain noise factors. This section proposes three constraints related to gait capturing environments to enhance the accuracy of gait anomaly detection.

### Key environmental challenges

**Fig. 9:**
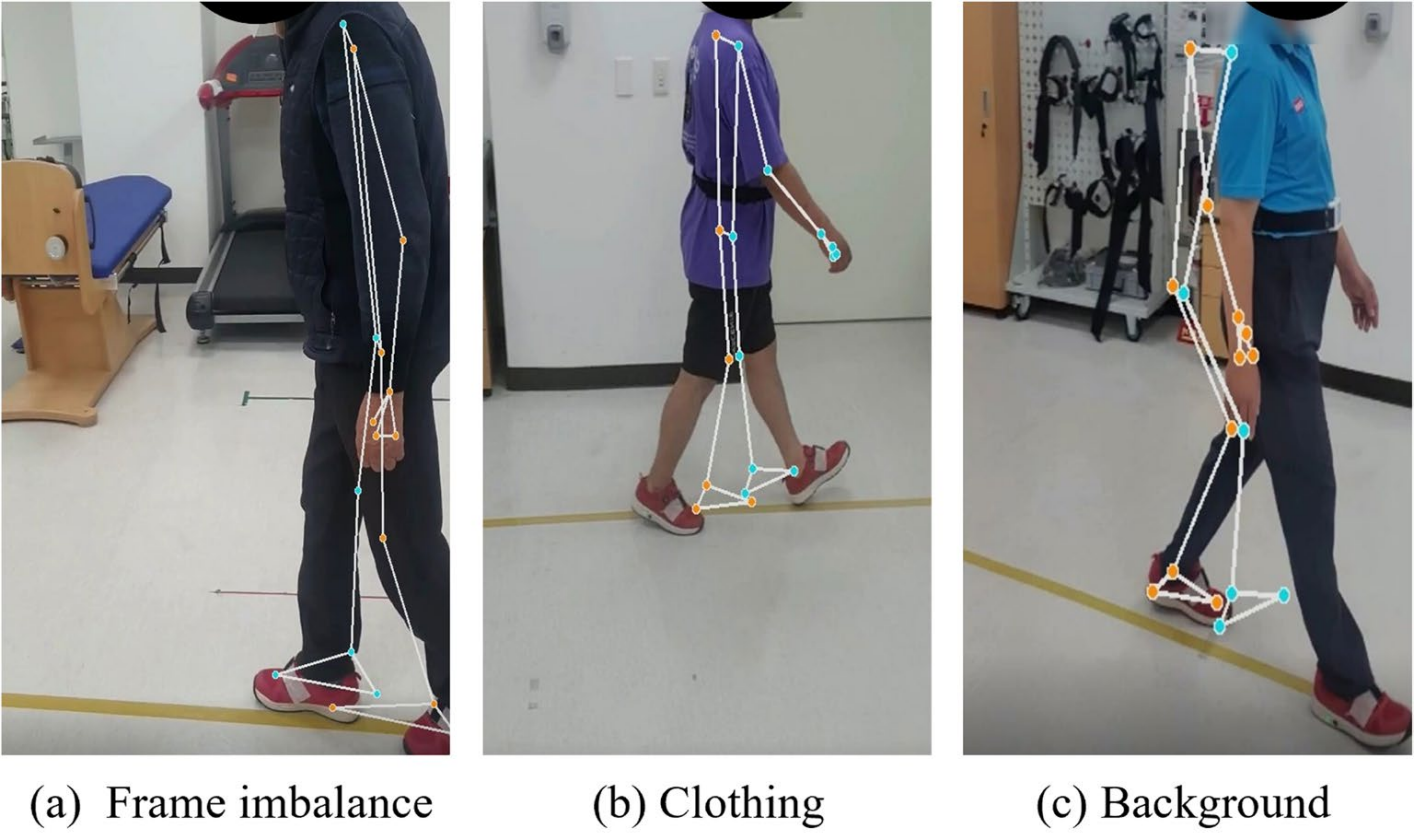
Three Mediapipe recognition errors.

Three primary environmental factors were identified as sources of recognition error in MediaPipe-based gait analysis:

- Frame imbalance-induced errors: occur when the pedestrian moves partially out of the camera frame, as shown in Fig. 9a.
- Clothing-induced errors: result from clothing that obscures body contours or blends into the background, leading to misidentification, as depicted in Fig. 9b.
- Background-induced errors: arise when MediaPipe confuses background objects with body joints, as seen in Fig. 9c.

Notably, the prevalence of recognition errors was 3% in normal gait data, 46% in sarcopenia (SA) data, and 18% in Parkinson’s disease (PD) data, highlighting the particular challenge posed by SA videos.

### A post-processing enhancement and data collection guidelines

To mitigate the aforementioned environmental challenges and improve the robustness of MediaPipe-based gait anomaly detection, this section presents a set of optimization strategies. These strategies include both post-processing methods for already-recorded gait videos and practical guidelines for future video acquisition. The proposed post-processing techniques aim to maximize data usability by recovering or refining noisy frames caused by issues such as clothing interference or frame imbalance. In parallel, we analyze common error patterns found in previously collected gait datasets and, based on this analysis, propose data collection guidelines to avoid such pitfalls in future recordings (e.g., camera angle, clothing contrast, background settings).

While this study focuses primarily on the analysis of existing noisy datasets, the effectiveness of our proposed data collection guidelines will be empirically evaluated in a follow-up study using newly acquired videos that adhere to these recommendations.

#### Post-processing enhancement: background removal

**Fig. 10:**
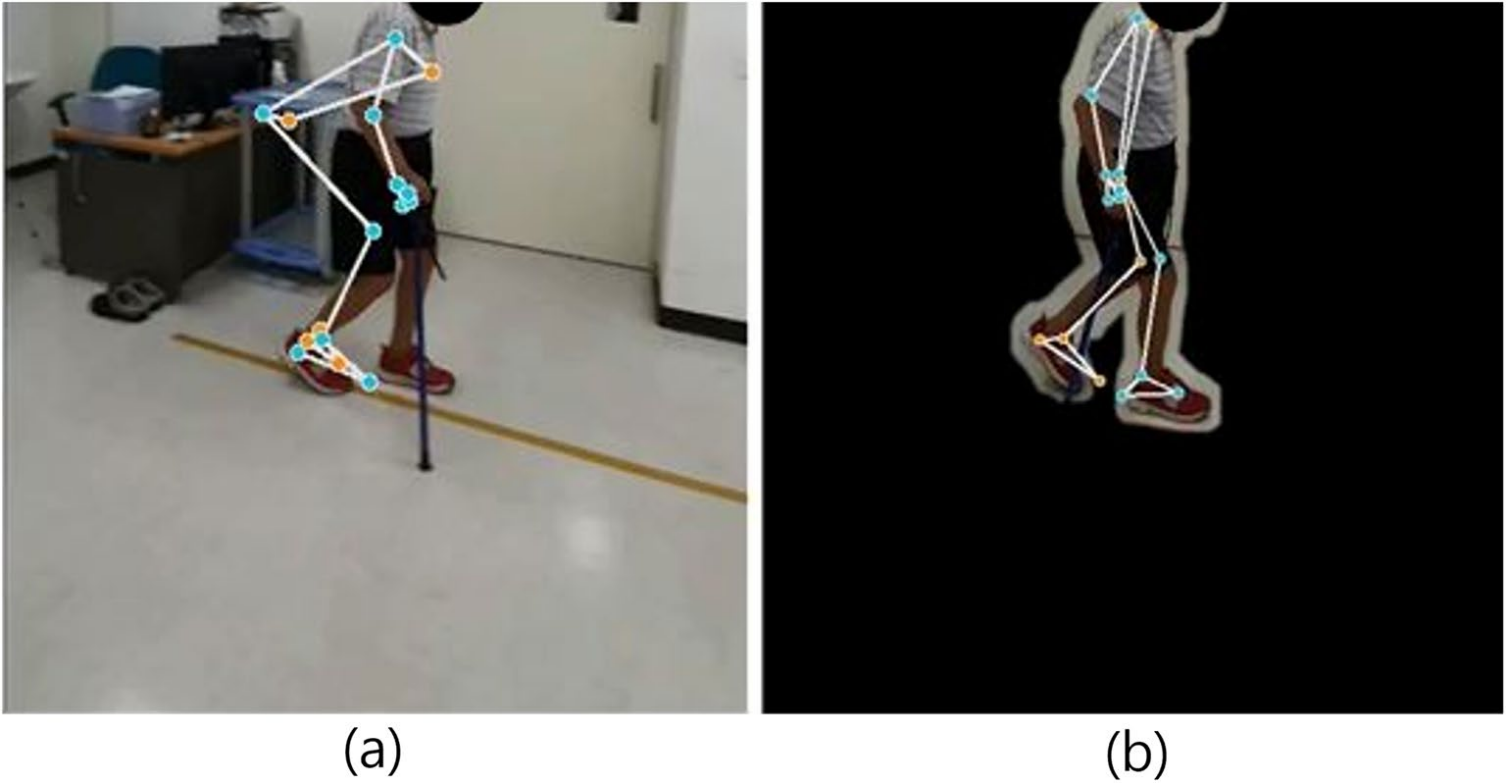
Before and after applying semantic segmentation.

Semantic segmentation^32^ was applied to mask background objects, as illustrated in Fig. 10, allowing MediaPipe to better isolate joint points. After background removal, usable data increased by 38% in normal videos, 33% in SA videos, but only 2% in PD videos. The lower improvement in PD data may reflect the disease’s inherent gait irregularity, which warrants further investigation.

**Fig. 11:**
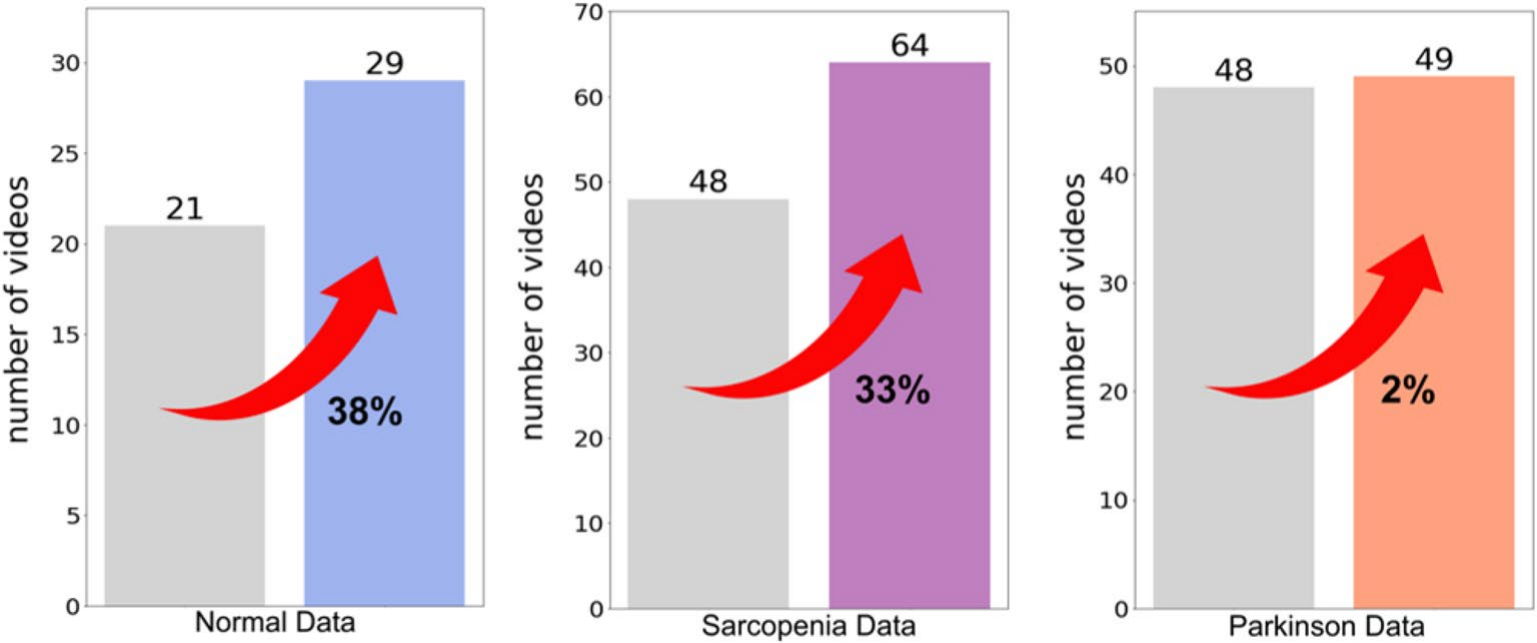
Data increase rate after semantic segmentation.

Figure 10a shows an image processed by MediaPipe before semantic segmentation, where certain background objects are mistakenly identified as joints of the target person. In contrast, Fig. 10b shows an image where the background around the target person has been removed using semantic segmentation, demonstrating that background removal can help MediaPipe better focus on the joint parts of the target.

Figure 11 illustrates the increase in usable data after applying semantic segmentation. For normal gait videos, usable frames increased by 38% (from 21 to 29). For sarcopenia (SA) patient videos, the improvement was 33% (from 48 to 64), while for Parkinson’s disease (PD) videos, the increase was only 2% (from 48 to 49).

This difference in improvement is attributable to the recording environments. SA videos were captured in indoor settings with cluttered backgrounds, where background removal significantly enhanced joint detection. In contrast, PD videos were recorded in clean hospital corridors with minimal visual noise, resulting in already stable joint tracking. As a result, semantic segmentation had a limited effect on improving PD data.

#### Guidelines for mitigating frame imbalance

MediaPipe can accurately extract gait information when the camera is fixed and both the pedestrian’s walking speed and the camera movement are consistent. However, irregular camera movement or pedestrian motion can result in joints moving out of frame, causing frame imbalance errors. To address frame imbalance errors caused by irregular camera or pedestrian movement, we employed YOLOv3^33^ to retrospectively analyze the spatial framing of subjects in existing gait videos. By estimating the proportion of each subject’s bounding box within the video frame, we identified optimal framing configurations that help ensure all key joints remain visible throughout the sequence. This analysis was used solely to derive guidelines for future data collection and was not involved in model training or inference.

**Fig. 12:**
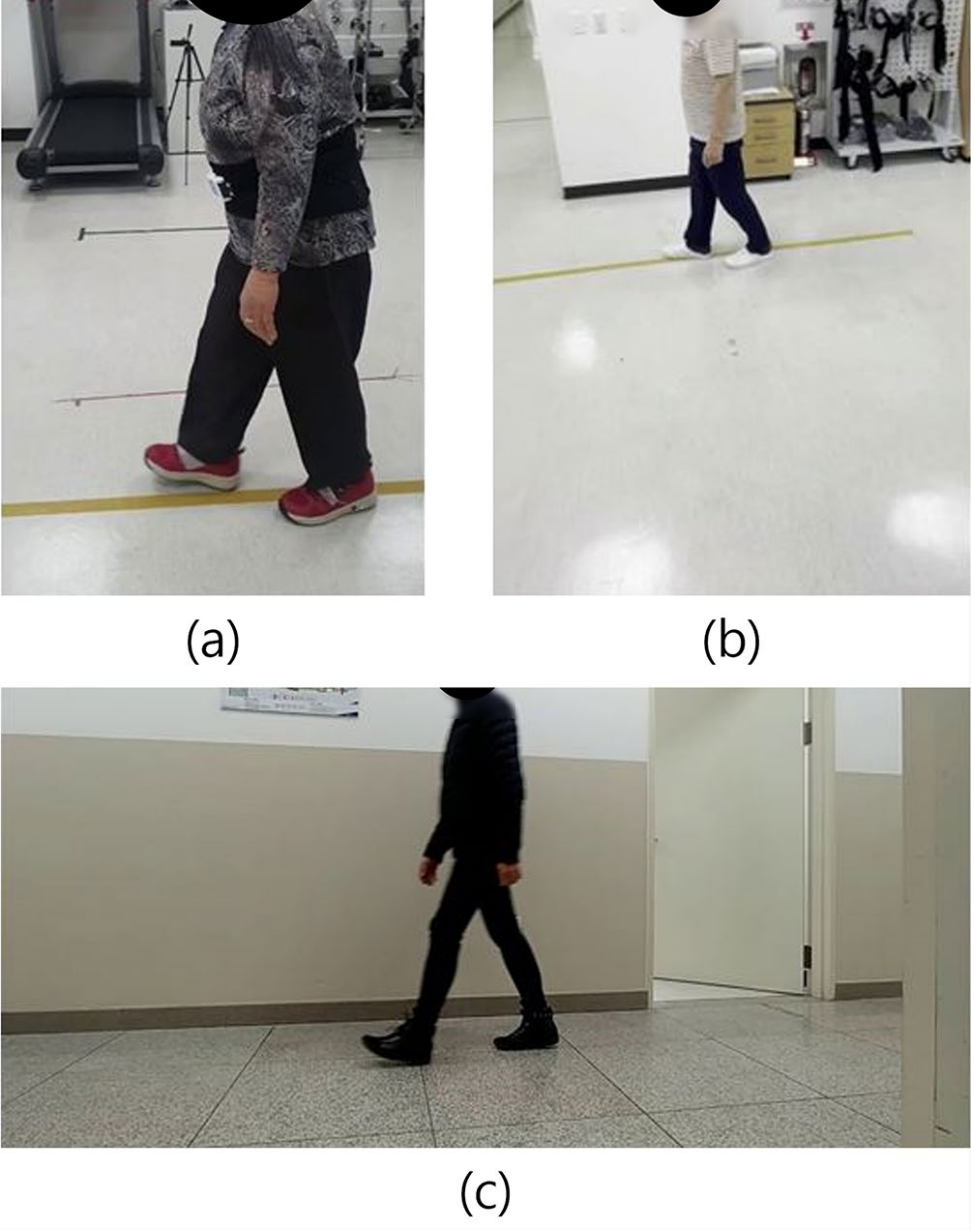
SA and PD lateral gait data.

The study focused on SA patient gait videos, which exhibited the greatest variability in frame margins compared to PD and normal gait data. As shown in Fig. 12, the target pedestrian can occupy varying portions of the frame, from large (a) to small (b), necessitating precise margin analysis.

**Fig. 13:**
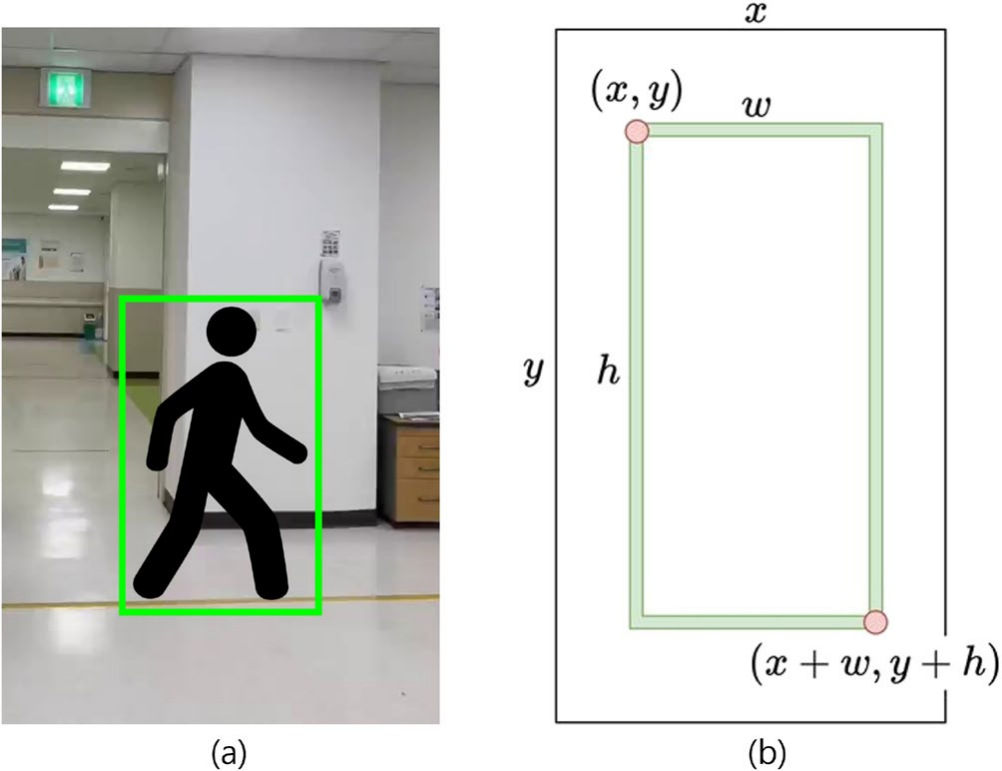
Frame of SA lateral gait video data applied with YOLO and coordinate values of a bounding box.

To analyze this variation, YOLOv3^33^ was used to extract bounding boxes across multiple videos, enabling empirical estimation of margin variability. The coordinates of the upper-left (*x*, *y*) and lower-right $(x + w, y + h)$ corners of each bounding box served as the basis for calculating ideal and maximum frame margin, as illustrated in Fig. 13. Note that YOLOv3 was not used during model inference or preprocessing; its use was limited strictly to offline margin analysis of existing data.

**Fig. 14:**
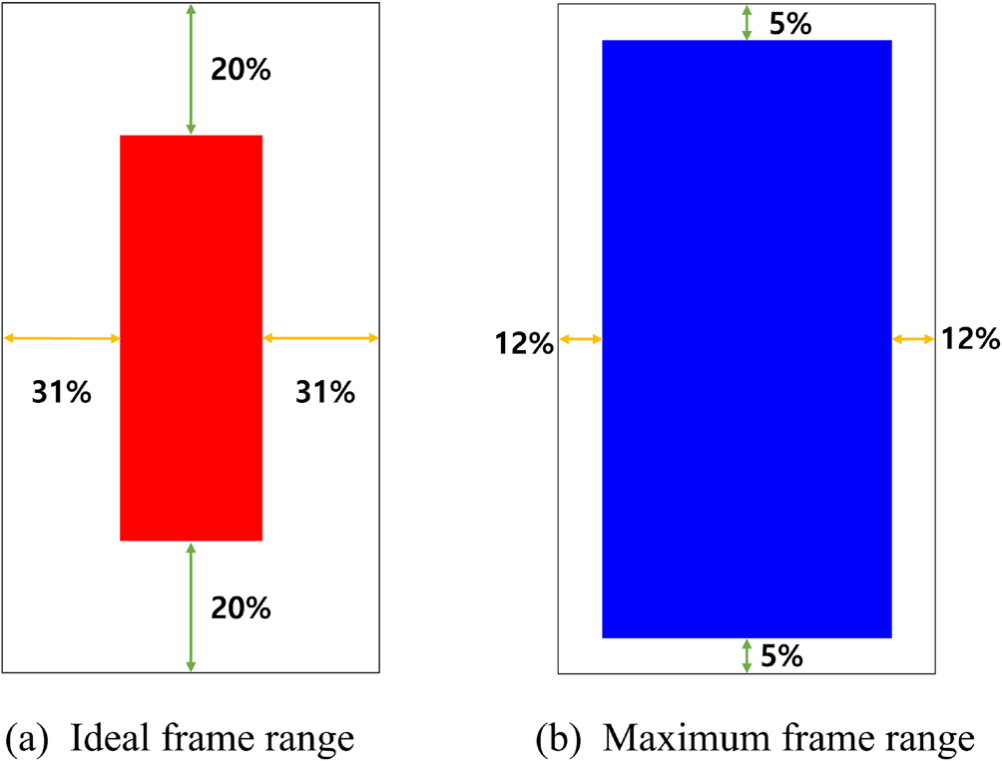
Range of frames for stable gait data tracking.

To visually guide data collection, Fig. 14 illustrates two framing configurations: (a) the ideal frame, which fully captures all five target joints with ample margins, and (b) the maximum allowable frame, which still preserves essential visibility for anomaly detection. These examples serve as practical references for recording setup, helping ensure consistent joint visibility and minimizing data loss due to poor framing.

**Table 9:** Recommended framing margins for gait video capture.

Margin direction	Ideal margin (%)	Maximum allowable margin (%)
Top–bottom	20	5
Left–right (sides)	31	12

To standardize video capture and improve joint visibility, Table 9 summarizes the recommended framing margins for gait recordings. These guidelines ensure that five key joint regions—right hip (RH), left hip (LH), right knee (RK), left knee (LK), and the nose–shoulder (NS) line—remain fully visible within the frame. To meet the recommended proportions, the camera distance and angle should be adjusted appropriately during video capture. Future data collection efforts should adhere to these framing instructions to enhance the consistency and diagnostic reliability of the gait anomaly detection model.

#### Guidelines for minimizing clothing-induced artifacts

To minimize artifacts caused by clothing interference, we recommend that participants wear plain, non-patterned clothing in colors that moderately contrast with the background. Adhering to this guideline can reduce pose estimation errors and enhance joint detection accuracy without requiring additional post-processing.

## Related work

This section reviews gait tracking research, covering sensor-based and image-based approaches, and discusses data refinement studies.

### Sensor-based gait tracking system

Kim et al. ^34^ proposed an inertial sensor-based gait model classifying seven gait phases with explainable AI, achieving 88.69% accuracy (osteopenia) and 93.75% (sarcopenia) using XGBoost and random forest, respectively. Vidya et al. ^35^ used a CNN-LSTM on EMD sensor data to classify Parkinson’s stages with 98.32% accuracy. Erdem et al. ^36^ applied smartwatch sensor data and machine learning (GPR, LSTM) to estimate stride and swing time, showing potential for clinical use. Paragliola et al. ^37^ combined CNN, LSTM, and DNN on shoe sensor data, achieving over 90% accuracy in Parkinson’s gait detection. Potluri et al. ^38^ used plantar pressure and IMU data with stacked LSTM to classify normal and abnormal gait types. Pach et al. ^39^ and Madhana et al. ^40^ used Kinect data and LSTM to classify Parkinson’s and hemiplegic gait, analyzing joint angles and step features.

Kim et al. ^17^ proposed a hybrid classification model that combines smart insole measurements with AI-based pose estimation to distinguish sarcopenia from control patients. Their approach used random forest, support vector machine, and artificial neural networks, achieving an accuracy range of 92–96% using pose estimation variables alone, and up to 100% when combined with insole data. This hybrid strategy demonstrated state-of-the-art performance and highlighted the potential of digital biomarkers for musculoskeletal diagnostics.

Compared to hybrid sensor–image approaches ^17^, our markerless video-based method achieved comparable or superior accuracy (97% for sarcopenia), without requiring wearable devices or supervised labels.

### Image-based gait tracking system

Pradhan et al. ^41^ used multi-segment foot kinematics to classify autism gait at 96.3% accuracy. Chen et al. ^42^ employed OpenPose and a random forest-LSTM model for sarcopenia gait phase analysis, achieving 90.9% accuracy. Jinnovart et al. ^43^ developed an RGB camera-based real-time abnormal gait system with RNN/LSTM, reaching 82.8% accuracy. Yang et al. ^44^ analyzed Parkinson’s gait from video using skeleton features, reaching 92.5% (random forest). Connie et al. ^45^ used AlphaPose to extract pose features, achieving 93% accuracy across views, identifying step length and arm swing as key indicators. Yunardi et al. ^46^ compared marker-based and markerless systems, finding marker-based methods better in joint errors, but markerless more robust in some exercises.

### Data refinement

Soundararajan et al. ^47^ applied a variational autoencoder and K-Means to preprocess multimodal data, improving Parkinson’s detection accuracy from 83% to 99.8%. Park et al. ^48^ used notch and high-pass filters on ECG signals, boosting CNN performance significantly. Khan et al. ^49^ enhanced color contrast and tuned YOLOv3 for traffic sign detection, achieving 98–100% accuracy.

### Differentiation and experimental design

Unlike prior works, which focus on classifying specific diseases, this study addresses generalized *abnormal gait detection* without disease constraints. It integrates *MediaPipe-based markerless tracking and an LSTM-autoencoder for anomaly detection*, enabling precise separation of normal and abnormal gait components. Notably, three environmental constraints were introduced to improve detection robustness, which prior image-based studies lack. The experimental design includes comparative evaluation under these constraints, providing evidence for enhanced practical applicability.

### Pose estimation under noisy conditions

Recent studies have demonstrated that pose estimation models are vulnerable to real-world image corruptions such as blur, compression, lighting changes, and occlusions. PoseBench^50^ systematically benchmarked several state-of-the-art models (e.g., ViTPose, HRNet, DEKR) under four corruption types, revealing significant performance degradation. These findings underscore the critical need for robustness in clinical environments, where such corruptions are common.

To address temporal inconsistency, Cotton et al.^51^ applied Kalman filter-based smoothing to improve joint trajectory stability. Zhang and Chen^52^ proposed a correntropy-based cost function (COI) to reduce the impact of outliers in pose estimation. Niculae et al.^53^ introduced artifact correction and adaptive bounding box strategies to improve pose estimation in highly compressed CCTV footage.

However, most of these approaches either require retraining or modify the pose estimation model itself, limiting their clinical scalability and real-time applicability. In contrast, our method enhances robustness through a lightweight and modular preprocessing pipeline that includes YOLO-based frame filtering, semantic segmentation for background removal, and clothing standardization. This data-centric strategy improves pose stability without altering model architecture, making it more practical for deployment in uncontrolled clinical or home environments.

### Gait-based disease classification

Prior works on disease classification from gait data have mainly relied on supervised learning and disease-specific labels. He et al.^18^ reconstructed 3D poses from 2D joint trajectories and used gait parameters (e.g., stride length, knee angle range) for Parkinson’s classification, achieving 93% accuracy. Seifallahi et al.^54^ combined OpenPose with CNNs to identify Alzheimer’s-related gait anomalies. Eguchi et al.^55^ distinguished Parkinson’s disease and spinocerebellar degeneration using a transformer-based deep neural network trained on 2D joint data.

While accurate, these approaches are often limited by the need for large annotated datasets and may not generalize well to unseen gait abnormalities. More recent studies have explored alternatives, such as MediaPipe with LSTM^56^, weak supervision^57^, and clinical rehabilitation analysis using 2D joint angles^58^.

Our study takes a fundamentally different approach by employing an unsupervised anomaly detection framework based on LSTM-autoencoders trained solely on normal gait. Rather than classifying specific diseases, we detect deviations from learned normal patterns, allowing broad applicability to various pathological gaits without relying on disease-specific annotations. This enhances the model’s adaptability and reduces labeling burden, making it a promising tool for early screening in diverse clinical and non-clinical settings.

## Discussion

### Limitations and future data expansion strategies

Although our framework achieved strong performance in detecting gait anomalies in sarcopenia and Parkinson’s disease patients, the dataset size remains relatively small, limiting the generalizability of the results. This is largely due to the difficulty of collecting gait data from elderly patients with mobility impairments.

To address this, we plan to expand the dataset through collaborations with multiple clinical centers. Future data collection will focus on including a wider range of gait patterns and disease severity levels, which will improve the robustness and applicability of our model in diverse clinical settings.

### Enhancing sensitivity via multi-joint prediction models

To improve sensitivity to subtle joint angle variations—particularly in anatomically challenging regions—we plan to explore multi-joint prediction models that leverage inter-joint relationships. Several recent studies have demonstrated promising results using surface electromyography (sEMG) and hybrid deep learning architectures.

Wan et al. ^59^ proposed an Informer-based model that continuously predicts hip, knee, and ankle angles using fused sEMG and temporal gait features, achieving correlation coefficients over 0.99 and MAE below $2^{\circ }$ for knees. Wang et al. ^60^ introduced a Dual Transformer Network capable of predicting both joint angles and torques from multi-channel sEMG, showing strong performance across all lower-limb joints.

Qin and Zhang ^61^ developed a Kalman-filter-enhanced neural network (SRUKFNN), significantly improving angle prediction accuracy (RMSE 0.78, correlation 0.99). Kumar et al. ^62^ demonstrated that physics-informed neural networks (PINNs) integrating biomechanical constraints outperform conventional ANN models in upper-limb joint prediction.

Inspired by these findings, we plan to integrate similar architectures into our framework to enhance detection robustness, particularly for subtle joint deviations. These approaches may enable earlier identification of gait anomalies and improve overall model generalization.

### Clinical interpretation and insights

This study benefited from the direct clinical involvement of a board-certified orthopedic surgeon, who is also a co-author. His domain expertise guided the selection of clinically relevant target diseases, the evaluation of gait anomalies at the joint level, and the interpretation of key findings—particularly the emphasis on knee joints as primary indicators. This collaboration ensures that the proposed framework aligns with real-world diagnostic needs and supports practical deployment in clinical settings.

## Conclusion

We proposed a fully markerless and contactless framework for detecting abnormal gait in orthopedic patients. The system uses MediaPipe for joint tracking and an unsupervised LSTM-autoencoder trained only on normal gait data. Without relying on wearable sensors or disease-specific labels, the model identifies deviations in gait patterns indicative of musculoskeletal conditions.

To address common sources of environmental noise, we introduced a preprocessing pipeline targeting frame imbalance, clothing artifacts, and background interference. These improvements increased usable gait data by up to 38%, enhancing detection consistency. The model achieved high accuracy—97% for sarcopenia and 88% for Parkinson’s disease. Joint-level analysis showed that the knees were the most sensitive indicators of abnormal gait, aligning with clinical expectations. This highlights the potential of our method for early orthopedic screening in real-world settings. We also proposed simple, practical guidelines for optimizing video capture and used a YOLO-based frame selection method to ensure joint visibility. These steps improved data quality without requiring changes to hardware.

Future work will focus on improving sensitivity to subtle joint variations by exploring multi-joint prediction models. We also plan to expand the dataset across multiple clinical sites to improve generalizability.

Overall, this study demonstrates that a robust, non-invasive gait analysis system is feasible and can contribute meaningfully to early diagnosis and monitoring in orthopedic care.

## Data Availability

The dataset used in this study is part of a cohort registered in Korea and is subject to restricted access within the country due to data protection regulations. However, fully anonymized data may be made available for replication purposes upon reasonable request to the corresponding author, provided that an approved anonymization procedure is implemented and ethically authorized. While public distribution is not currently permitted, we are exploring the possibility of sharing representative anonymized samples or synthetic gait data in future versions to support reproducibility.

## References

[1] Bakator, M. & Radosav, D. Deep learning and medical diagnosis: A review of literature. *Multimodal Technol. Interact.* **2**, 47 (2018).

[2] Xu, Y. et al. Deep learning predicts lung cancer treatment response from serial medical imaging. *Clin. Cancer Res.* **25**, 3266–3275 (2019). 31010833 10.1158/1078-0432.CCR-18-2495 PMC6548658

[3] Chen, H., Engkvist, O., Wang, Y., Olivecrona, M. & Blaschke, T. The rise of deep learning in drug discovery. *Drug Discov. Today* **23**, 1241–1250 (2018). 29366762 10.1016/j.drudis.2018.01.039

[4] Liu, T., Siegel, E. & Shen, D. Deep learning and medical image analysis for covid-19 diagnosis and prediction. *Annu. Rev. Biomed. Eng.* **24**, 179–201 (2022). 35316609 10.1146/annurev-bioeng-110220-012203

[5] Hussain, M., Koundal, D. & Manhas, J. Deep learning-based diagnosis of disc degenerative diseases using MRI: A comprehensive review. *Comput. Electr. Eng.* **105**, 108524 (2023).

[6] Humayun, M., Khalil, M. I., Almuayqil, S. N. & Jhanjhi, N. Z. Framework for detecting breast cancer risk presence using deep learning. *Electronics* **12**, 403 (2023).

[7] McKinney, S. M. et al. International evaluation of an AI system for breast cancer screening. *Nature* **577**, 89–94 (2020). 31894144 10.1038/s41586-019-1799-6

[8] Ting, D. S. W. et al. Development and validation of a deep learning system for diabetic reopathy and related eye diseases using real images from multiethnic populations with diabetes. *JAMA* **318**, 2211–2223 (2017). 29234807 10.1001/jama.2017.18152 PMC5820739

[9] D’Angelo, T. et al. Artificial intelligence, machine learning and deep learning in musculoskeletal imaging: Current applications. *J. Clin. Ultrasound* **50**, 1414–1431 (2022). 36069404 10.1002/jcu.23321

[10] Abdullah, S. S. & Rajasekaran, M. P. Automatic detection and classification of knee osteoarthritis using deep learning approach. *La Radiol. Med.* **127**, 398–406 (2022). 10.1007/s11547-022-01476-7 35262842

[11] McCoy, D. et al. Convolutional neural network-based automated segmentation of the spinal cord and contusion injury: Deep learning biomarker correlates of motor impairment in acute spinal cord injury. *Am. J. Neuroradiol.* **40**, 737–744 (2019). 30923086 10.3174/ajnr.A6020 PMC7048524

[12] Chen, C. P. et al. Sagittal plane loading response during gait in different age groups and in people with knee osteoarthritis. *Am. J. Phys. Med. Rehabil.* **82**, 307–312 (2003). 12649658 10.1097/01.PHM.0000056987.33630.56

[13] Valderrabano, V. et al. Gait analysis in ankle osteoarthritis and total ankle replacement. *Clin. Biomech.* **22**, 894–904 (2007). 10.1016/j.clinbiomech.2007.05.003 17604886

[14] Neal, B. S. et al. *Phys. Ther. Sport* **43**, 36–42 (2020). 32066107 10.1016/j.ptsp.2020.02.004

[15] Viswakumar, A., Rajagopalan, V., Ray, T. & Parimi, C. Human gait analysis using openpose. In *2019 Fifth International Conference on Image Information Processing (ICIIP)*. 310–314 (IEEE, 2019).

[16] Hii, C. S. T. et al. Marker free gait analysis using pose estimation model. In *2022 IEEE 20th Student Conference on Research and Development (SCOReD)*. 109–113 (IEEE, 2022).

[17] Kim, S., Kim, H. S. & Yoo, J.-I. Sarcopenia classification model for musculoskeletal patients using smart insole and artificial intelligence gait analysis. *J. Cachexia Sarcopenia Muscle* (2023). 10.1002/jcsm.13356. 10.1002/jcsm.13356 PMC10751435 37884824

[18] He, R. et al. A novel multi-level 3D pose estimation framework for gait detection of Parkinson’s disease using monocular video. *Front. Bioeng. Biotechnol.* **12**, 1520831. 10.3389/fbioe.2024.1520831 (2024). 39764149 10.3389/fbioe.2024.1520831 PMC11700975

[19] Roubenoff, R. & Hughes, V. A. Sarcopenia: Current concepts. *J. Gerontol. Ser. A Biol. Sci. Med. Sci.* **55**, M716–M724 (2000). 11129393 10.1093/gerona/55.12.m716

[20] Kalia, L. V. & Lang, A. E. Parkinson’s disease. *Lancet* **386**, 896–912 (2015). 25904081 10.1016/S0140-6736(14)61393-3

[21] Perry, J. & Burnfield, J. *Gait Analysis: Normal and Pathological Function* (CRC Press, 2024).

[22] Lugaresi, C. et al. Mediapipe: A framework for building perception pipelines. *arXiv preprint* arXiv:1906.08172 (2019).

[23] Cao, Z., Simon, T., Wei, S.-E. & Sheikh, Y. Realtime multi-person 2D pose estimation using part affinity fields. In *Proceedings of the IEEE Conference on Computer Vision and Pattern Recognition*. 7291–7299 (2017).

[24] Yu, Y., Si, X., Hu, C. & Zhang, J. A review of recurrent neural networks: LSTM cells and network architectures. *Neural Comput.* **31**, 1235–1270 (2019). 31113301 10.1162/neco_a_01199

[25] Provotar, O. I., Linder, Y. M. & Veres, M. M. Unsupervised anomaly detection in time series using LSTM-based autoencoders. In *2019 IEEE International Conference on Advanced Trends in Information Theory (ATIT)*. 513–517 (IEEE, 2019).

[26] Deb, S., Singh, H. P., Kumar, S. & Kanbur, S. M. Morphology and metallicity of the small magellanic cloud using rrab stars. *Mon. Not. R. Astron. Soc.* **449**, 2768–2783. 10.1093/mnras/stv358 (2015).

[27] Malhotra, P.et al. LSTM-based encoder-decoder for multi-sensor anomaly detection (2016). arXiv:1607.00148.

[28] Wei, Y. et al. LSTM-autoencoder-based anomaly detection for indoor air quality time-series data. *IEEE Sens. J.* **23**, 3787–3800 (2023).

[29] Nguyen, H. D., Tran, K. P., Thomassey, S. & Hamad, M. Forecasting and anomaly detection approaches using LSTM and LSTM autoencoder techniques with the applications in supply chain management. *Int. J. Inf. Manag.* **57**, 102282 (2021).

[30] Liu, P. et al. Arrhythmia classification of LSTM autoencoder based on time series anomaly detection. *Biomed. Signal Process. Control* **71**, 103228 (2022).

[31] Roy, M., Majumder, S., Halder, A. & Biswas, U. ECG-net: A deep LSTM autoencoder for detecting anomalous ECG. *Eng. Appl. Artif. Intell.* **124**, 106484 (2023).

[32] Long, J., Shelhamer, E. & Darrell, T. Fully convolutional networks for semantic segmentation. In *Proceedings of the IEEE Conference on Computer Vision and Pattern Recognition*. 3431–3440 (2015). 10.1109/TPAMI.2016.2572683 27244717

[33] Redmon, J. & Farhadi, A. Yolov3: An incremental improvement. *arXiv preprint* arXiv:1804.02767 (2018).

[34] Kim, J.-K., Bae, M.-N., Lee, K., Kim, J.-C. & Hong, S. G. Explainable artificial intelligence and wearable sensor-based gait analysis to identify patients with osteopenia and sarcopenia in daily life. *Biosensors* **12**, 167 (2022). 35323437 10.3390/bios12030167 PMC8946270

[35] Vidya, B. & Sasikumar, P. Parkinson’s disease diagnosis and stage prediction based on gait signal analysis using EMD and CNN-LSTM network. *Eng. Appl. Artif. Intell.* **114**, 105099 (2022).

[36] Erdem, N. S., Ersoy, C. & Tunca, C. Gait analysis using smartwatches. In *2019 IEEE 30th International Symposium on Personal, Indoor and Mobile Radio Communications (PIMRC Workshops)*. 1–6 (IEEE, 2019).

[37] Paragliola, G. & Coronato, A. Gait anomaly detection of subjects with Parkinson’s disease using a deep time series-based approach. *IEEE Access* **6**, 73280–73292 (2018).

[38] Potluri, S., Ravuri, S., Diedrich, C. & Schega, L. Deep learning based gait abnormality detection using wearable sensor system. In *2019 41st Annual International Conference of the IEEE Engineering in Medicine and Biology Society (EMBC)*. 3613–3619 (IEEE, 2019). 10.1109/EMBC.2019.8856454 31946659

[39] Pachón-Suescún, C. G., Pinzón-Arenas, J. O. & Jiménez-Moreno, R. Abnormal gait detection by means of LSTM. *Int. J. Electr. Comput. Eng. (2088-8708)* **10** (2020).

[40] Madhana, K., Jayashree, L. & Perumal, K. System for classification of human gaits using markerless motion capture sensor. *J. Enabling Technol.* **17**, 41–53 (2023).

[41] Pradhan, A., Chester, V. & Padhiar, K. Classification of autism and control gait in children using multisegment foot kinematic features. *Bioengineering* **9**, 552 (2022). 36290520 10.3390/bioengineering9100552 PMC9598184

[42] Chen, Y.-C. et al. Towards deep learning-based sarcopenia screening with body joint composition analysis. In *2021 IEEE International Conference on Image Processing (ICIP)*. 3807–3811 (IEEE, 2021).

[43] Jinnovart, T., Cai, X. & Thonglek, K. Abnormal gait recognition in real-time using recurrent neural networks. In *2020 59th IEEE Conference on Decision and Control (CDC)*. 972–977 (IEEE, 2020).

[44] Yang, Y. et al. A video-based method to classify abnormal gait for remote screening of Parkinson’s disease. In *2021 40th Chinese Control Conference (CCC)*. 3357–3362 (IEEE, 2021).

[45] Connie, T. et al. Pose-based gait analysis for diagnosis of Parkinson’s disease. *Algorithms* **15**, 474 (2022).

[46] Yunardi, R. T., Sardjono, T. A. & Mardiyanto, R. Motion capture system based on rgb camera for human walking recognition using marker-based and markerless for kinematics of gait. In *2023 IEEE 13th Symposium on Computer Applications & Industrial Electronics (ISCAIE)*. 262–267 (IEEE, 2023).

[47] Soundararajan, R. et al. Deeply trained real-time body sensor networks for analyzing the symptoms of Parkinson’s disease. *IEEE Access* **10**, 63403–63421 (2022).

[48] Park, Y., Yun, I. D. & Kang, S.-H. Preprocessing method for performance enhancement in cnn-based STEMI detection from 12-lead ECG. *IEEE Access* **7**, 99964–99977 (2019).

[49] Khan, J. A., Chen, Y., Rehman, Y. & Shin, H. Performance enhancement techniques for traffic sign recognition using a deep neural network. *Multimed. Tools Appl.* **79**, 20545–20560 (2020).

[50] Ma, S., Zhang, J., Cao, Q. & Tao, D. Posebench: Benchmarking the robustness of pose estimation models under corruptions. *arXiv preprint *arXiv:2406.14367 (2024).

[51] Cotton, R. J. et al. Improved trajectory reconstruction for markerless pose estimation. *arXiv preprint *arXiv:2303.02413 (2023). 10.1109/EMBC40787.2023.10340745 38083280

[52] Zhang, Q. & Chen, B. Robust pose estimation based on maximum correntropy criterion. In *Artificial Intelligence Applications and Innovations*(Maglogiannis, I., Macintyre, J. & Iliadis, L. Eds.). 555–566 (Springer, 2021).

[53] Niculae, A., Catruna, A., Cosma, A., Rosner, D. & Radoi, E. Gait recognition from highly compressed videos (2024). arXiv:2404.12183.

[54] Seifallahi, M., Farrell, B., Galvin, J. E. & Ghoraani, B. Human pose estimation and gait analysis with convolutional neural networks for Alzheimer’s disease detection. In *Big Data VI: Learning, Analytics, and Applications* (Markopoulos, P. P. Ed.). Vol. 13036. 130360L. 10.1117/12.3013776. (International Society for Optics and Photonics/SPIE, 2024).

[55] Eguchi, K. et al. Feasibility of differentiating gait in Parkinson’s disease and spinocerebellar degeneration using a pose estimation algorithm in two-dimensional video. *J. Neurol. Sci.* **464**, 123158. 10.1016/j.jns.2024.123158 (2024). 39096835 10.1016/j.jns.2024.123158

[56] Khalil, H., Saad, A. M. S. E. & Khairuddin, U. Diagnosis of cerebellar ataxia based on gait analysis using human pose estimation: A deep learning approach. In *2022 IEEE-EMBS Conference on Biomedical Engineering and Sciences (IECBES)*. 201–206. 10.1109/IECBES54088.2022.10079396 (2022).

[57] Gholami, M. et al. Automatic labeling of Parkinson’s disease gait videos with weak supervision. *Med. Image Anal.* **89**, 102871. 10.1016/j.media.2023.102871 (2023). 37480795 10.1016/j.media.2023.102871

[58] Nishizawa, K. et al. Evaluation of the clinical utility of a gait analysis system using pose estimation techniques in physical therapy. In *2024 SICE International Symposium on Control Systems (SICE ISCS)*. 107–112. 10.23919/SICEISCS60954.2024.10505757 (2024).

[59] Wan, R., Zhu, Z., Cui, G. & Chen, S. Continuous prediction of multi-joint angles based on informer model using novel fused features. *Meas. Sci. Technol.* **36**, 015709. 10.1088/1361-6501/ad9ca1 (2024).

[60] Wang, Z. et al. Dual transformer network for predicting joint angles and torques from multi-channel emg signals in the lower limbs. *IEEE J. Biomed. Health Inform.* 1–13. 10.1109/JBHI.2025.3555255 (2025). 10.1109/JBHI.2025.3555255 40146650

[61] Qin, P. & Zhang, J. Lower limb joint angle prediction using square root untraced Kalman neural network based on SEMG signal. In *Proceedings of the Third International Conference on Electronic Information Engineering, Big Data, and Computer Technology (EIBDCT 2024)*. Vol. 13181. 131818S. 10.1117/12.3031024 (SPIE, 2024).

[62] Kumar, R., Muthukrishnan, S. P., Kumar, L. & Roy, S. Predicting multi-joint kinematics of the upper limb from EMG signals across varied loads with a physics-informed neural network (2023). arXiv:2312.09418.

